# Physiological roles of embryonic microglia and their perturbation by maternal inflammation

**DOI:** 10.3389/fncel.2025.1552241

**Published:** 2025-04-07

**Authors:** Tsukasa Shimamura, Masashi Kitashiba, Kazutaka Nishizawa, Yuki Hattori

**Affiliations:** Department of Anatomy and Cell Biology, Graduate School of Medicine, Nagoya University, Nagoya, Japan

**Keywords:** brain, development, maternal immune activation, maternal inflammation, microglia, neurodevelopmental disorder, neuron, psychiatric disorder

## Abstract

The interplay between the nervous and immune systems is well documented in the context of adult physiology and disease. Recent advances in understanding immune cell development have highlighted a significant interaction between neural lineage cells and microglia, the resident brain macrophages, during developmental stages. Throughout development, particularly from the embryonic to postnatal stages, diverse neural lineage cells are sequentially generated, undergo fate determination, migrate dynamically to their appropriate locations while maturing, and establish connections with their surroundings to form neural circuits. Previous studies have demonstrated that microglia contribute to this highly orchestrated process, ensuring the proper organization of brain structure. These findings underscore the need to further investigate how microglia behave and function within a broader framework of neurodevelopment. Importantly, recent epidemiological studies have suggested that maternal immune activation (MIA), triggered by various factors, such as viral or bacterial infections, environmental stressors, or other external influences, can affect neurogenesis and neural circuit formation, increasing the risk of neurodevelopmental disorders (NDDs) in offspring. Notably, many studies have revealed that fetal microglia undergo significant changes in response to MIA. Given their essential roles in neurogenesis and vascular development, inappropriate activation or disruption of microglial function may impair these critical processes, potentially leading to abnormal neurodevelopment. This review highlights recent advances in rodent models and human studies that have shed light on the behaviors and multifaceted roles of microglia during brain development, with a particular focus on the embryonic stage. Furthermore, drawing on insights from rodent MIA models, this review explores how MIA disrupts microglial function and how such disturbances may impair brain development, ultimately contributing to the onset of NDDs.

## 1 Introduction

Microglia serve as specialized immune cells of the central nervous system (CNS), which comprises the brain, the spinal cord, and the retina. Their cellular characteristics and functions have been elucidated through decades of research. Microglia were initially recognized as innate immune cells that serve as the first line of defense against damage-associated agents, as well as against bacterial and viral infections within the CNS (Ajami et al., 2018; Picard et al., 2021; Waltl and Kalinke, 2022; Thorsdottir et al., 2019; Kent and Miron, 2024; Borst et al., 2021). Historically, their roles were thought to be primarily restricted to pathological conditions, including aging, chronic stress, and neurodegenerative diseases such as Alzheimer’s disease, Parkinson’s disease and multiple sclerosis, where they mediate neuroinflammatory responses (Streit et al., 2020; Tynan et al., 2010; Venegas et al., 2017; Leng and Edison, 2021; Chen and Colonna, 2021). However, increasing evidence has highlighted their crucial functions in normal physiological states: microglia facilitate neuronal differentiation, regulate synaptic organization, and preserve the CNS environment through the clearance of cellular debris and apoptotic cells (Wake et al., 2009; Paolicelli et al., 2011; Parkhurst et al., 2013; Shemer et al., 2015; Sierra et al., 2014; Wilton et al., 2023).

While the diverse functions of microglia in the adult and postnatal stages have been extensively studied, increasing evidence has suggested that microglia perform various functions during embryonic stage. During embryonic period, diverse neural lineage cells are sequentially generated, undergo fate determination, migrate dynamically to their appropriate locations and connect with surrounding cells, thereby establishing neural circuits. These processes are tightly regulated through complicated mechanisms, enabling the construction of a well-organized neocortex (Rakic, 1974; Nadarajah et al., 2001; Tabata et al., 2012; Stoufflet et al., 2023). Recent studies have shed light on the critical roles of microglia in regulating neurogenesis (Cunningham et al., 2013; Arno et al., 2014; Hattori and Miyata, 2018), glial production (Nakanishi et al., 2007; Marsters et al., 2020; Sherafat et al., 2021), neuronal circuit formation (Squarzoni et al., 2014), and blood vessel formation (Fantin et al., 2010) during early developmental stages. Given that microglia play essential roles in various aspects of neurogenesis and vascular development, their unnecessary activation or disruption may adversely affect their proper functioning and lead to aberrant neurogenesis. Recent epidemiological studies have suggested that maternal immune activation (MIA) is associated with an increased risk for neurodevelopmental disorders (NDDs) in the offspring (Han et al., 2021; Kim et al., 2024). Notably, many studies have revealed that the properties of fetal microglia change in response to MIA. For example, MIA results in increased expression of proinflammatory cytokine genes and elevated cytokine production by microglia (Schaafsma et al., 2017; O’Loughlin et al., 2017; Loayza et al., 2023).

This review highlights the latest findings on microglial colonization and their diverse roles on surrounding cells in the developing brain, with a particular focus on their effects during the embryonic stage. Furthermore, it explores how disturbances in the interaction between microglia and their surrounding cells caused by MIA might hinder brain development, potentially resulting in psychiatric disorders or neurodegenerative diseases.

## 2 Microglial ontology and colonization of the developing brain

**FIGURE 1:**
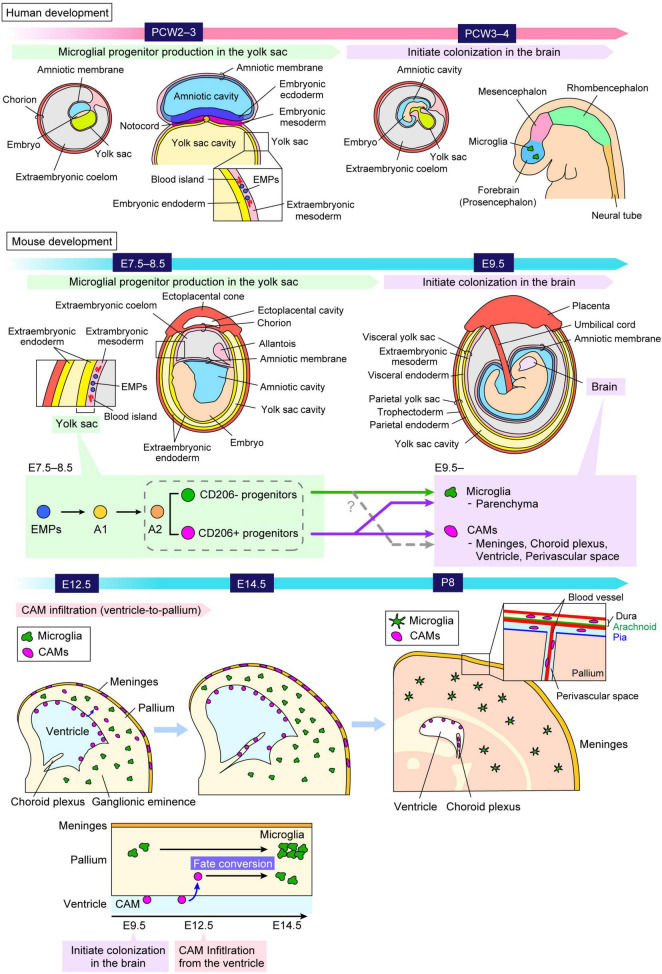
Ontology and distribution of “brain macrophages.” A schematic illustrates the process by which microglial progenitors colonize the brain both in humans and mice. Microglia and CAMs originate from the same progenitors, EMPs, which arise in the extraembryonic mesoderm of the yolk sac around PCW2–3 in humans and E7.5–8.5 in mice. Microglial progenitors differentiate from EMPs into microglia through the A1 and A2 states, and thereafter these cells migrate into the brain around at PCW3–4 in humans and around E9.5 in mice. A previous study reported that A2 progenitors can be divided into two distinct cell populations, characterized by the expression levels of CD206, a specific marker for CAMs (Utz et al., 2020). Another study using fate-mapping demonstrated that CD206^+^ progenitors retain the potential to differentiate into both CAMs and microglia, indicating that microglia are not exclusively derived from CD206^–^ progenitors but also arise from early-committed CD206^+^ progenitors (Masuda et al., 2022a). Furthermore, it was revealed that a subset of microglia is derived from intraventricular CAMs, which frequently infiltrate the pallium at E12.5 in mice, indicating that microglia consist of at least two distinct populations with different colonization pathways (Hattori et al., 2023). The precise mechanisms governing when, where, and how these cells commit to their respective fates, as well as the factors influencing their migration into the brain parenchyma, remain to be fully elucidated. CAM, central nervous system-associated macrophage; E, embryonic day; EMP, erythromyeloid progenitor; P, postnatal day; PCW, postconceptional week.

Neural lineage cells, including other glial cells (astrocytes and oligodendrocytes), originate from the neuroectoderm. In contrast, microglia are derived from erythromyeloid progenitors (EMPs), which are generated in the blood islands of the extraembryonic mesoderm in the yolk sac during embryogenesis (Ginhoux et al., 2010; Alliot et al., 1999; Menassa and Gomez-Nicola, 2018; Kierdorf et al., 2013; Prinz et al., 2021; Figure 1).

Microglia are maintained independently from the circulatory monocytes and sustain themselves through self-renewal throughout life under physiological conditions (Askew et al., 2017; Réu et al., 2017; Nikodemova et al., 2015; Askew and Gomez-Nicola, 2018). In the developing cortex of mice, once microglia are seeded in the embryonic brain at embryonic day (E) 9.5, the number of microglia increases through proliferation until the postnatal stage and peaks two weeks after birth (Alliot et al., 1999; Nikodemova et al., 2015; Askew and Gomez-Nicola, 2018; Askew et al., 2017; Navascues et al., 2000). Their cell population size is maintained by slow homeostatic proliferation of preexisting mature cells *in situ* in a clonal fashion (Barry-Carroll et al., 2023). A stable density of microglia is sustained through their low proliferative capacity until adulthood (Dalmau et al., 2003; Ginhoux et al., 2010; Arnoux et al., 2013; Nikodemova et al., 2015; Askew et al., 2017). Microglia exhibit long lifespans of several months to years in both mice (Goldmann et al., 2016; Askew et al., 2017) and humans (Réu et al., 2017). In the adult mouse brain, microglia undergo slow self-renewal, with an estimated daily turnover rate of 0.79% (Askew et al., 2017). In humans, microglia renew at a median rate of 28% per year, with an average cell age of 4.2 years. Over a lifetime, more than 96% of the microglial population is replaced (Réu et al., 2017).

In mice, EMPs are generated at E7.5–8.5, and subsequently, microglial precursors immigrate into the brain at E9.5 before the formation of blood–brain barrier (Ginhoux et al., 2010; Stremmel et al., 2018; Figure 1). During embryogenesis, EMPs give rise to an intermediate population of immature macrophages, termed A1, which further develop into a premacrophage progenitor population, termed A2 (Prinz et al., 2021; Masuda et al., 2022b). These progenitors migrate extensively along the developing blood vessels and begin infiltrating the neural tube around E9.5 (Ginhoux et al., 2010; Stremmel et al., 2018). They differentiate into microglia and central nervous system-associated macrophages (CAMs), a distinct type of brain macrophage, located at the interface between the vascular system and the brain parenchyma (Dalmau Gasull et al., 2024). A previous study reported that A2 progenitors can be divided into two distinct cell populations, characterized by the expression levels of CD206 (Mannose Receptor C-type 1, MRC1), a specific marker for CAMs (Utz et al., 2020). They proposed a model that a “brain macrophage population” may segregate early in development, with distinct progenitor types giving rise to CAMs (CD206^+^) and microglia (CD206^–^), respectively (Utz et al., 2020). On the other hand, the study using fate-mapping demonstrated that CD206^+^ progenitors retain the potential to differentiate into both CAMs and microglia, indicating that microglia are not exclusively derived from CD206^–^ progenitors but also arise from early-committed CD206^+^ progenitors (Masuda et al., 2022a). In addition, another study revealed that some microglia are derived from intraventricular CAMs, which frequently infiltrate the pallium at E12.5 in mice, indicating that microglia consist of at least two distinct populations with different colonization pathways (Hattori et al., 2023).

Human microglial development has similarities to mouse microglia. In humans, developmental microglia first appear in the extraembryonic mesoderm of the yolk sac at approximately postconceptional week (PCW) 2–3 (Janossy et al., 1986; Nogales, 1993; Popescu et al., 2019; Park et al., 2020; Figure 1). These cells are later detectable in the forebrain/telencephalon around PCW3–4 before the onset of large-scale neurogenesis and neuronal migration (Monier et al., 2007; Verney et al., 2010; Menassa and Gomez-Nicola, 2018; Menassa et al., 2022). A recent study reported that microglia exhibit remarkable heterogeneity during development and acquire immune responsiveness from PCW10 (Kracht et al., 2020). A study based on post-mortem human brain samples, ranging from PCW3 to 75 years of age, demonstrated that, after colonization, particularly around PCW9 (the embryonic-fetal transition), the density of microglia fluctuates significantly, exhibiting wave-like patterns of proliferation followed by apoptosis (Menassa et al., 2022). Throughout the embryonic, fetal, and postnatal periods, as well as after birth, the microglial population undergoes different cycles of expansion and apoptosis-driven refinement. This process stabilizes during childhood and is maintained in adulthood and older ages through gradual self-renewal (Menassa et al., 2022).

## 3 Microglial distribution in the fetal brain

**FIGURE 2:**
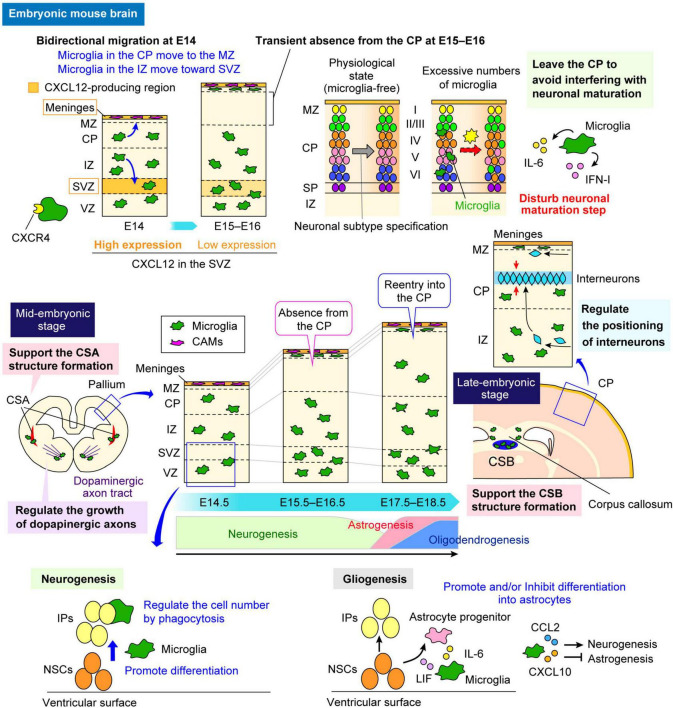
Multifaceted functions of microglia in the embryonic brain. The multifaceted functions of microglia in the embryonic brain are highlighted. A schematic illustrates how microglia alter their distribution in a stage-dependent manner during cerebral cortex development in mice and the roles they play in specific regions. During early embryonic stages, microglia are homogeneously distributed in the pallium. However, they transiently disappear from the CP between E15 and E16 before reentering around E17 (center panel). In regions where microglia are abundant, such as the VZ and the SVZ, they regulate neurogenesis and gliogenesis through cytokine and chemokine production (sections “5.1 Physiological and MIA-induced effects on neurogenesis” and “5.4 Physiological and MIA-induced effects on gliogenesis”). Conversely, the transient absence of microglia from the CP between E15 and E16 is crucial for the neuronal maturation, as microglia adjust their positioning to avoid interference (section “5.2 Physiological and MIA-induced effects on neuronal circuit formation”). In addition, microglia contribute to neuronal circuit formation by modulating the growth of dopaminergic axons and axon tracts in the corpus callosum (section “5.2 Physiological and MIA-induced effects on neuronal circuit formation”). They also play a role in interneuron positioning and migration (section “5.3 Physiological and MIA-induced effects on interneurons”). Furthermore, microglia support structural integrity in the CSA at E14 and CSB at E18 (section “3 Microglial distribution in the fetal brain”). CAM, central nervous system-associated macrophage; CCL2, C-C motif chemokine ligand 2; CP, cortical plate; CSA, cortico-striato-amygdalar boundary; CSB, cortico-septal boundary; CXCL10, C-X-C chemokine ligand 10; CXCL12, C-X-C chemokine ligand 12; CXCR4, C-X-C chemokine receptor 4; IFN-I, type I interferon; IL-6, interleukin-6; IP, intermediate progenitor; IZ, intermediate zone; LIF, leukemia inhibitory factor; MZ, marginal zone; NSC, neural stem cell; SP, subplate; SVZ, subventricular zone; VZ, ventricular zone.

After settling in the brain, microglia change their distribution in a stage-dependent manner in the developing cerebral cortex (Figure 2). In mice, microglia are homogenously distributed throughout the developing cortex, from the ventricular zone (VZ), where neural stem cells (NSCs) reside, to the cortical plate (CP), where mature neurons start to accumulate around E13–E14. However, at E15–E16, microglia temporarily disappear from the CP, and by E17, they reappear within the CP (Swinnen et al., 2013; Hattori, 2021). The study demonstrated that C-X-C chemokine ligand 12 (CXCL12), released from the meninges and the subventricular zone (SVZ), regulates microglial bidirectional migration, which occurs at E14 in the mouse embryonic brain (Hattori et al., 2020). At E14, microglia initially positioned in the intermediate zone (IZ) migrate toward the SVZ, whereas the cells in the CP move toward the meninges and thereafter accumulate in the marginal zone (MZ); thus, microglia are expelled from the CP from E15 to E16.

During embryogenesis, microglia most actively and extensively migrate throughout the pallium at E14, as demonstrated through live imaging (Swinnen et al., 2013; Hattori and Miyata, 2018). From the late embryonic to postnatal stages, microglia preferentially colonize the VZ/SVZ, which is the neurogenic region of the telencephalon in rodents and primates (Antony et al., 2011; Cunningham et al., 2013; Arno et al., 2014, Squarzoni et al., 2014). In regions where microglia are abundant, they have been shown to play essential roles in various developmental processes, including neurogenesis (Cunningham et al., 2013; Arno et al., 2014; Hattori and Miyata, 2018), gliogenesis (Nakanishi et al., 2007; Marsters et al., 2020; Sherafat et al., 2021), and interneuron migration guidance (Squarzoni et al., 2014)—processes that will be further discussed in later sections (Figure 2). During embryonic development, microglia have also been reported to support structural integrity and stability in other brain regions (Lawrence et al., 2024; Figure 2). Lawrence et al. (2024) reported that fetal cortical boundaries, including the cortico-striato-amygdalar boundary (CSA) and the cortico-septal boundary (CSB), are particularly sensitive to morphogenetic stress, which induces a microglial state similar to that of postnatal axon-tract-associated microglia (ATMs). This state plays a novel role at these boundaries by preventing the development of cavitary lesions, partly through secreted phosphoprotein 1 (SPP1)-mediated phagocytosis of fibronectin 1. Microglia expressing SPP1 are involved in the swift repair of lesions, underscoring their protective functions in preserving the fetal brain from physiological morphogenetic stress and damage.

While microglia perform various functions in regions where they are abundant, their temporary absence from certain areas suggests a strategic mechanism to avoid interfering with the surrounding cells, ensuring proper development. The reason for microglial temporary absence at E15–E16 from the CP has been revealed to be the bidirectional migration of microglia at E14 (Hattori et al., 2020). Specifically, microglia exhibit chemotaxis through their expression of C-X-C chemokine receptor 4 (CXCR4) in response to CXCL12, which is strongly expressed in intermediate progenitors (IPs) in the SVZ at E14 and in the meninges from E13 onward. The transient absence of microglia from the CP at E15–E16 has been shown to be important for the proper neuronal subtype maturation, suggesting that, by temporarily relocating from the CP, microglia may create a microenvironment that facilitates the differentiation and maturation of neuronal subtypes (Hattori et al., 2020; Figure 2).

In humans, neural progenitors proliferate within the VZ around gestational week (GW) 8–28 (PCW6–26), and then migrate toward the CP around GW9–38 (PCW7–36) (Bystron et al., 2008; Marin, 2016; Menassa and Gomez-Nicola, 2018; Matuleviciute et al., 2023). Upon reaching the CP, they differentiate into distinct neuronal subtypes and synaptogenesis is initiated around GW26 (PCW24) and form neural network (Tau and Peterson, 2010; Marin, 2016). A series of post-mortem studies report that microglia appear in the ventral prosencephalon (which later develops into the telencephalon and diencephalon) from PCW4 (Monier et al., 2007; Verney et al., 2010), but a more recent study reported that microglia reach the forebrain around PCW3 (Menassa et al., 2022). The early arrival of microglia to the brain precedes the onset of pivotal processes of human cortical development, such as neurogenesis, neuronal migration, and gliogenesis (Menassa and Gomez-Nicola, 2018). As the CP expands around GW8 (PCW6), microglia populate the VZ, SVZ, IZ, subplate (SP), and the MZ (Kostovic and Rakic, 1990; Rezaie et al., 2005; Monier et al., 2007). Microglia within the IZ are thought to proliferate around GW8–12 (PCW6–10). Around GW9–13 (PCW7–11), proliferative microglia align along the SP-CP boundary and remain there, avoiding infiltration into the CP until the late third trimester (Kostovic and Rakic, 1990; Rezaie et al., 2005; Monier et al., 2007). By GW18 (PCW16), intermediate microglia migrate radially within the CP and eventually differentiate into ramified gray matter microglia (Monier et al., 2007; Verney et al., 2010).

Research on microglial development has advanced significantly in mice, providing detailed insights into colonization routes and underlying mechanisms. While studies in humans have been more challenging due to limited accessibility of brain samples, important findings have highlighted shared aspects of microglial development between rodents and humans. Although multiple routes of microglial brain colonization have been identified in mice, the exact mechanisms in humans remain to be fully elucidated. Further research is needed to clarify the similarities and differences in microglial colonization patterns between humans and mice.

## 4 Modeling MIA in neurodevelopmental disorder studies

Epidemiological data suggest that genetic risk provides a foundation upon which other factors may precipitate or enhance the risk of NDDs (Song and Chung, 2010; Zawadzka et al., 2021). One of those other risk factors is prenatal infection. MIA can be triggered by various factors, including bacterial and viral infections, allergies, and autoimmune diseases. These factors activate the mother’s immune system, leading to an inflammatory response during pregnancy, a phenomenon commonly referred to as MIA. Epidemiological and preclinical studies for humans suggest that MIA increases the risk of NDDs, such as schizophrenia, bipolar disorder, autism spectrum disorders (ASD), attention deficit/hyperactivity disorder (ADHD), cerebral palsy, developmental delay, cognitive dysfunction, and depression (Knuesel et al., 2014; Bergdolt and Dunaevsky, 2019; Meyer, 2019). It remains unclear whether microglial dysfunction directly causes NDDs in humans. However, in certain neurodevelopmental disorders, such as colony-stimulating factor 1 receptor (CSF1R)-related pediatric leukoencephalopathy—a condition caused by homozygous or heterozygous mutations in the *Csf1r* gene—microglia are permanently absent, leading to pronounced brain structural abnormalities, including white matter defects and cerebellar hypoplasia (Oosterhof et al., 2019). Children with these mutations exhibit cognitive, motor, and sensory impairments, as well as severe neurodevelopmental delays (Oosterhof et al., 2019).

One limitation of these human studies is their reliance on an observational design, which prevents a full understanding the causal relationship between MIA and symptoms of NDDs. Therefore, animal models are important for understanding the role of the immune system in neurodevelopmental perturbations under MIA conditions. To model MIA in rodents, polyinosinic-polycytidylic acid [Poly(I:C)], a synthetic analog of double-stranded RNA, is administered to the dam to simulate viral double-stranded RNA (Smith et al., 2007; Meyer, 2014; Hayes et al., 2022). For example, the administration of Poly(I:C) to pregnant mice elicits ASD-related behaviors in the offspring, including deficits in sociability and repetitive behaviors (Smith et al., 2007; Malkova et al., 2012; Haddad et al., 2020; Andoh et al., 2019; Schaafsma et al., 2017). Another common approach involves the use of lipopolysaccharide (LPS), a bacterial cell wall component, administered via maternal injection to trigger an immune response. In rats, offspring born to LPS-treated dams, such as juveniles and young adults, exhibit reduced social preference and exploratory behaviors (Oskvig et al., 2012).

Emerging evidence from animal studies shows that MIA during pregnancy impacts placental function and disrupts neuronal circuit formation in the fetal brain, resulting in abnormal behavioral traits characteristic of NDDs in offspring (Knuesel et al., 2014; Bergdolt and Dunaevsky, 2019; Meyer, 2019). Intraperitoneal injection of Poly(I:C) or LPS into a pregnant rodent significantly elevates proinflammatory cytokine levels and biological marker levels in maternal serum, placenta, amniotic fluid, fetal brain and cerebrospinal fluid (Zengeler et al., 2023; Woods et al., 2023; Hameete et al., 2021; Petrova et al., 2024; Cui et al., 2020). On the other hand, animal studies have demonstrated that the administration of proinflammatory cytokines, specifically interleukin (IL)-6 or IL-17, is sufficient to induce NDD-like phenotypes in offspring, as blocking these cytokines during MIA induction ameliorates these phenotypes (Smith et al., 2007; Wu et al., 2017; Choi et al., 2016). However, the detailed mechanisms by which MIA or elevated cytokines during pregnancy are transmitted to the fetus and how they affect the fetal brain remain poorly understood.

Given that microglia play essential roles in neurogenesis, neuronal circuit formation, and blood vessel development, their unnecessary activation or disruption may adversely affect their proper functioning and lead to aberrant neurogenesis. Several reports suggest that MIA causes changes in the microglial transcriptome that persist into adulthood in offspring (Matcovitch-Natan et al., 2016; Hayes et al., 2022; Ostrem et al., 2024). Furthermore, previous studies have reported that the properties of fetal microglia change in response to MIA. For example, MIA results in increased expression of proinflammatory cytokine genes and cytokine production by microglia (Schaafsma et al., 2017; O’Loughlin et al., 2017; Loayza et al., 2023). Notably, a recent study using MIA model mice induced by Poly(I:C) demonstrated that MIA-induced transcriptomic changes in neuronal cell types were reduced/abolished by genetic depletion of microglia in *Csf1r*^–/–^ mice (Ostrem et al., 2024). They also identified lasting transcriptional changes in fetal microglia that persist postnatally (Ostrem et al., 2024). These findings suggest that the absence of microglia has a significant effect on the transcriptomes of cells in the developing cortex, including pyramidal neurons, interneurons, and vascular component cells.

## 5 Multifaceted functions of embryonic microglia and their disturbance in MIA

**FIGURE 3:**
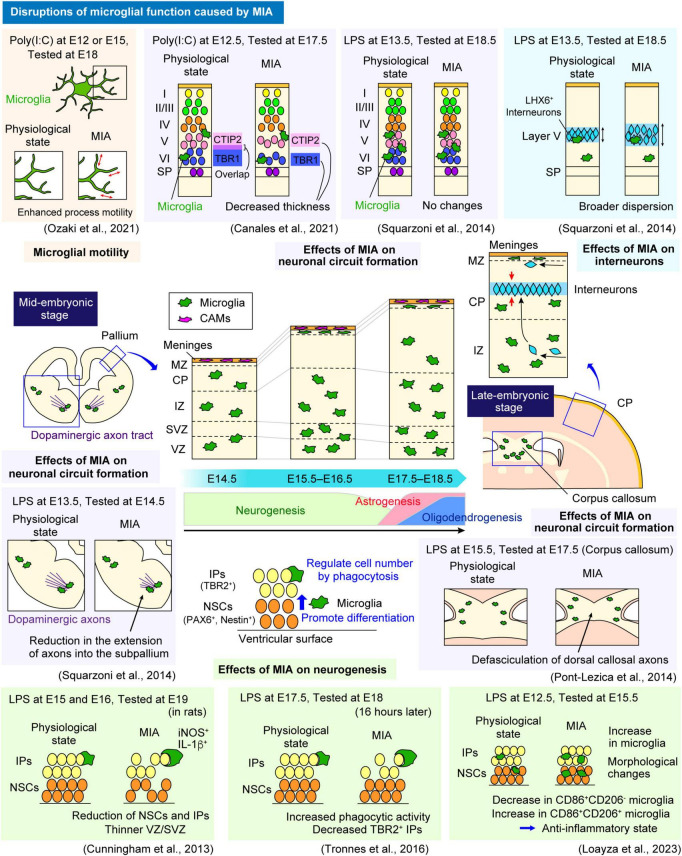
MIA-induced effects on microglial function in the embryonic stage. A schematic summarizes previous studies investigating how MIA, induced by LPS or Poly(I:C), disrupts microglial function and its impact on surrounding neural lineage cells during embryonic development in rodent models. In the context of neurogenesis (section “5.1 Physiological and MIA-induced effects on neurogenesis”), MIA alters the state of microglia responsible for regulating the differentiation and number of NSCs and IPs, leading to a reduction in their population. Regarding its impacts on neuronal circuit formation (section “5.2 Physiological and MIA-induced effects on neuronal circuit formation”), MIA induces defasciculation of dorsal callosal axons in the corpus callosum and impairs dopaminergic axon extension in the subpallium. The effects of MIA on pyramidal neurons remain debated, with some studies reporting no significant changes, while another study has observed alterations in the number of CTIP2^+^ and TBR1^+^ neurons. In the process of interneuron migration guidance (section “5.3 Physiological and MIA-induced effects on interneurons”), MIA disrupts the positioning of LHX6^+^ interneurons in layer V of the CP, resulting in a broader dispersion of the LHX6^+^ population. Regarding microglial motility (section “5.5 Physiological and MIA-induced effects on microglial mobility and motility”), MIA has been reported to increase microglial process motility during the embryonic stage. CAM, central nervous system-associated macrophage; CP, cortical plate; CSA, cortico-striato-amygdalar boundary; CSB, cortico-septal boundary; CTIP2, COUP-TF-interacting protein 2; CXCL10, C-X-C chemokine ligand 10; CXCL12, C-X-C chemokine ligand 12; CXCR4, C-X-C chemokine receptor 4; IL-1β, interleukin-1 beta; iNOS, inducible nitric oxide synthase; IP, intermediate progenitor; IZ, intermediate zone; LHX6, LIM Homeobox 6; LPS, lipopolysaccharide; MIA, maternal immune activation; MZ, marginal zone; NSC, neural stem cell; PAX6, Paired box gene 6; Poly(I:C), polyinosinic-polycytidylic acid; SP, subplate; SVZ, subventricular zone; TBR1, T-box brain transcription factor 1; TBR2, T-box brain transcription factor 2; VZ, ventricular zone.

Microglia constitute approximately 10% of all cells in the adult brain, whereas these cells make up only 0.5%–1.0% of those in the embryonic brain in mice (Alliot et al., 1999; Hammond et al., 2019; Hattori et al., 2020). Despite their relatively low numbers in the embryonic brain, microglia display high motility, actively moving their cell bodies and extending filopodia to conduct extensive surveillance (Swinnen et al., 2013; Smolders et al., 2017; Hattori et al., 2020). Such mobility enables them to interact with various surrounding cells, including neural lineage cells such as NSCs, neural progenitor cells (NPCs), and neurons, and vascular components such as endothelial cells and pericytes (Hattori et al., 2022). Indeed, embryonic microglia play pivotal roles in brain development by interacting with these cell types (Figure 2). Concurrently, an increasing number of studies, mainly conducted in rodent models, has suggested that MIA affects the properties and functions of microglia in the embryonic stage. In this section, we review previous studies that investigated the roles of fetal brain microglia in key developmental events and how their dysfunction can lead to disruptions in brain development, focusing on each event in sequence (Figure 3).

### 5.1 Physiological and MIA-induced effects on neurogenesis

During the embryonic stage, microglia tend to colonize the VZ/SVZ, which is a neurogenic region rich in NPCs, radial glial cells (RGCs), IPs, migrating NPCs or immature neurons in both rodents and primates (Antony et al., 2011; Cunningham et al., 2013; Arno et al., 2014; Squarzoni et al., 2014). Studies using CSF1R null mice, in which microglia are absent, have raised the possibility that microglia influence neurogenesis in the embryonic brain (Chitu and Stanley, 2017; Chitu et al., 2022). Other studies have reported that CSF1R-deficient mice exhibit abnormal brain structures, including reduced brain size, atrophy of the olfactory bulb, and enlargement of the lateral ventricles (Nandi et al., 2012; Erblich et al., 2011). In another study using *Csf1r^flox/flox^* mice under the control of the cytomegalovirus (CMV) promoter, which allows for the conditional depletion of microglia, a reduction in TBR2 (T-box brain transcription factor 2)^+^ IPs within the SVZ was observed during the middle and late embryonic stages (Arno et al., 2014). The reduction in TBR2^+^ IPs due to microglial depletion was also supported by another study. Consistent with this, experiments in mice revealed that the microglial depletion via liposomal clodronate injection into the ventricles at E13 led to a decrease in TBR2^+^ IPs and an increase in PAX6 (Paired box gene 6)^+^ NSCs at E15 (Hattori and Miyata, 2018; Figure 2). *In vitro*, when microglia were added to cultured NPCs and cocultured for 24 h, there was an increase in the number of TBR2^+^ IPs and a decrease in the number of PAX6^+^ immature NSCs, compared with those in the absence of microglia (Hattori and Miyata, 2018). These findings suggest that microglia facilitate neurogenesis by promoting the exit of NPCs from the cell cycle, and promote the differentiation of NSCs into IPs. Moreover, a recent study using brain organoids containing cells with microglia-like phenotypes and functions (iMicro) demonstrated that iMicro cells limited NPC proliferation and promoted axonogenesis (Park et al., 2023), suggested that microglia promote the differentiation of NPCs.

Microglia also regulate the cell population size of NPCs by phagocytosis. A previous study reported that microglia engulf NPCs to reduce their number in the developing rat brain (Cunningham et al., 2013). Inducing a proinflammatory state in microglia through MIA via the administration of LPS to the dam at E15 and E16 resulted in a reduction of both PAX6^+^ NSCs and TBR2^+^ IPs in the VZ/SVZ, leading to a thinner VZ/SVZ in the late embryonic period (E19) in rats (Cunningham et al., 2013; Figure 3). Furthermore, microglia expressing inducible nitric oxide synthase (iNOS) and interleukin-1 beta (IL-1β), indicative of an inflammatory state with enhanced phagocytic activity, increased in the VZ/SVZ in MIA model. In contrast, microglial inactivation by minocycline, induction of an anti-inflammatory state by doxycycline, and microglial depletion by liposomal clodronate increased the number of PAX6^+^ and TBR2^+^ cells in the VZ/SVZ. Additionally, they demonstrated that microglia not only engulf apoptotic cells but also live NPCs and mature neurons, suggesting that microglia actively modulate the cell population rather than merely eliminating dead cells (Cunningham et al., 2013). Consistent with these findings, another study revealed that microglia exhibiting phagocytic features in the VZ/SVZ establish close connections with TBR2^+^ IPs in mice (Tronnes et al., 2016). In a MIA model induced by LPS administration to the dam at E17.5, microglial phagocytic activity was increased, leading to a reduction in TBR2^+^ IPs at E18 (16 h later) (Tronnes et al., 2016). The same team as Cunningham et al. (2013) reported that microglia closely interact with the scaffold of RGCs and extensively contact NPCs within the proliferative zones of both rhesus monkey and rodent fetuses (Barger et al., 2019). A unique subset, termed “periventricular microglia,” interacts closely with mitotic NPCs in the VZ near the lateral ventricle during late cortical neurogenesis, as daughter cells delaminate and migrate toward the SVZ. While microglia interact with NPCs in both species, their density is significantly greater in the proliferative zones of rhesus monkey fetuses. These findings suggest that microglia serve as structural modulators by remodeling the proliferative zones by supporting NPC migration from the ventricle and promoting their delamination (Barger et al., 2019). Another study revealed that embryonic neurogenesis involves a critical interaction between microglia and NPCs. AT-rich interactive domain-containing protein 1A (ARID1A), an epigenetic regulator in microglia, disrupts chromatin remodeling and alters the expression of regulatory elements that control microglial state transitions. This disturbance impairs microglial release of proteoglycan 3 (PRG3), a factor essential for self-renewal and differentiation of NPCs. In turn, the loss of PRG3 disrupts the Wnt/β-catenin signaling pathway in NPCs by affecting their receptor low-density lipoprotein receptor-related protein 6 (LRP6), leading to abnormal neuronal development and autism-like behaviors later in life (Su et al., 2024).

Loayza et al. (2023) reported that MIA affects the regulation of neurogenesis by microglia. MIA induced by LPS at E12.5 in mice led to a robust increase in fetal (E15.5) and neonatal microglia (postnatal day (P) 1) in neurogenic periventricular regions, and also induced a dramatic change of microglia morphology, enlarged soma sizes, and retraction of processes (Loayza et al., 2023). Homeostatic microglia at E15.5 and P4 are heterogeneous, consisting of CD86^+^/CD206^–^ and CD86^+^/CD206^+^ subpopulations, whereas maternal LPS administration significantly reduces the CD86^+^/CD206^–^ population but increases the CD86^+^/CD206^+^ subpopulation, suggesting that MIA shifts microglia toward an anti-inflammatory or alternative state (Loayza et al., 2023). In addition, they also showed that MIA resulted in a robust increase in Ki67^+^/Nestin^+^ NSCs and TBR2^+^ NPCs in the neurogenic zone of neonatal (P1) mice, highlighting its impact on neurogenesis regulation by microglia.

Overall, microglia play a crucial role in maintaining the NPC pool by promoting differentiation and/or sustaining NPC numbers during brain development. When this regulatory balance is disrupted by MIA, the size and differentiation state of the NPC pool become dysregulated.

### 5.2 Physiological and MIA-induced effects on neuronal circuit formation

The effects of microglia on neurons in the postnatal brain have been widely reported, with studies showing that microglia are involved in key processes of neural network formation, such as regulating neuronal numbers, synaptic development and refinement, myelin maturation, synaptic transmission, and neuronal excitability (Hammond et al., 2018; Prinz et al., 2021; Miyamoto et al., 2016; Weinhard et al., 2018; Sipe et al., 2016; Parkhurst et al., 2013), whereas fewer studies have examined their role during embryonic development. Nevertheless, the functions of embryonic microglia on neural circuit formation are gradually becoming clearer.

In the studies by Squarzoni et al. (2014) various models were comprehensively investigated in the embryonic stage in mice, including microglial depletion models such as transient blockade of the CSF1R signaling pathway through injections of anti-CSF1R antibody at E6.5 and E7.5 and *Pu.*1^–/–^ mice, which lack the transcription factor PU.1, also known as SPI1, and consequently fail to generate all myeloid cells, including microglia. However, no significant effects on pyramidal neurons have been reported in these microglia depletion models. For example, the E18.5 somatosensory cortices of brains from microglia-depleted mice revealed no apparent effects on cortical layering or axonal invasion, indicating that microglial absence did not affect these processes (Figure 2). On the other hand, microglial depletion affects the laminar positioning of subsets of neocortical interneurons and the outgrowth of dopaminergic axons in the subpallium (Squarzoni et al., 2014; Figure 2).

On the other hand, a recent study reported that microglia regulate neuronal circuit formation by interacting with immature neurons (Cserép et al., 2022). Cserép et al. (2022) demonstrated that microglial processes form specialized contacts with developing neurons not only during adult and postnatal stages but also in the embryonic stage (Cserép et al., 2022). Moreover, deletion of purinergic receptor P2Y, G-protein coupled 12 (P2RY12) in microglia disrupted neuronal precursor proliferation and led to abnormal cortical development, suggesting that these purinergic junctions are crucial for microglia to monitor immature neurons and regulate neurodevelopment (Cserép et al., 2022).

Microglia not only exert their influences by interacting with their surroundings but also leave the area in a timely manner to avoid interference with neighboring cells. Hattori et al. (2020) reported that microglia migrate bidirectionally within the midembryonic developing cortex, temporarily vacating the CP at E15–E16. When postmigratory neurons in the CP were exposed to excessive number of microglia, either *in vivo* (through the overexpression of attractant factors or microglia transplantation) or *in vitro* (through coculture with microglia), they failed to properly express neuronal subtype-associated transcription factors, exhibiting altered expression patterns of neuron markers. Specifically, these neurons tended toward reduced expression of deep-layer neuronal marker genes and increased expression of typical upper-layer neuronal marker genes. The authors also demonstrated that IL-6 and type I interferon (IFN-I) from microglia disrupt gene expression in these neurons, indicating that the absence of microglia from the CP at E15–E16 is essential for proper neuronal differentiation (Hattori et al., 2020).

An increasing number of studies have investigated how MIA affects microglial function at the postnatal stage, particularly in relation to neuronal circuit formation. For example, late-gestational LPS exposure (E15.5–E17.5) in mice triggers a proinflammatory response in fetal microglia, and offspring exhibit reduced home cage activity, lower anxiety, and impaired learning (Schaafsma et al., 2017). Treating MIA [induced by Poly(I:C)] offspring with a CSF1R inhibitor followed by microglial repopulation reversed the MIA-induced abnormal behavior, suggesting that MIA affects microglial-synaptic interactions and/or pruning functions (Ikezu et al., 2021). In a Poly(I:C)-induced MIA model, impaired microglial synapse engulfment led to synaptic excess in the CA3 region of the hippocampus in the postnatal brain (Andoh et al., 2019).

However, less is known about how microglial changes influence neuronal circuit formation during the embryonic stage. Transcriptional profiling at E17.5 in a mouse MIA model induced by LPS at E15.5 revealed downregulation of genes critical for nervous system development (Pont-Lezica et al., 2014; Figure 3). This study also demonstrated that MIA led to defasciculation of dorsal callosal axons, highlighting the essential role of microglia in promoting neurite development and corpus callosum formation (Pont-Lezica et al., 2014). The study by Squarzoni et al. (2014) reported that MIA induced by intraperitoneal injections of LPS at E13.5 and analyzed at E18.5 had no significant effects on pyramidal neurons in the somatosensory cortices, suggesting that prenatal immune activation may not have a significant impact on neuronal circuit formation. On the other hand, another study using an MIA model induced by administering Poly(I:C) to the dam at E12.5 examined the distribution of neuronal subtypes in the CP at E17.5 (Canales et al., 2021). This study reported a reduced overlap in the positioning of TBR1 (T-box brain transcription factor 1)-positive and CTIP2 (COUP-TF-interacting protein 2)-positive neurons under MIA conditions, along with a decrease in both cell density and the thickness of the TBR1^+^ and CTIP2^+^ regions. Squarzoni et al. (2014) also reported that MIA induced by LPS impaired the extension of dopaminergic axons in the subpallium.

Together, since only a limited number of studies have explored microglial alterations during the embryonic stage and their effects on neuronal circuit development, further investigation is required to clarify their impacts.

### 5.3 Physiological and MIA-induced effects on interneurons

As differentiation progresses, NPCs undergo radial migration toward the basal surface as differentiation progresses (Rakic, 1974; Nadarajah et al., 2001; Tabata et al., 2012; Stoufflet et al., 2023). In contrast, inhibitory interneurons are predominantly produced in the ganglionic eminence (GE) and migrate tangentially to reach the neocortex (Marín et al., 2001; Tamamaki et al., 2003; Toudji et al., 2023). Recent studies reported that MIA affects interneuron development. For example, Vasistha et al. (2020) reported that MIA induced by injecting Poly(I:C) to the dam at E9.5 disrupts cortical gamma-amino butyric acid (GABA)-producing interneurons in mice. This disruption affects precursor proliferation, neuroblast migration, positioning, and maturation at E14.5 and E17.5 (Vasistha et al., 2020). Specific interneuron subtypes are affected, resulting in reduced cell numbers, altered distributions, and changes in morphology and properties. These subtype-specific vulnerabilities vary depending on the developmental stage and contribute to cognitive impairments in affected offspring (Vasistha et al., 2020).

Embryonic microglia have been reported to regulate the positioning of interneurons migrating from the GE to the pallium in the developing cortex (Squarzoni et al., 2014; Figure 2). Using an MIA model induced by intraperitoneal LPS injection at E13.5, the study revealed that microglia regulate the entry and positioning of LHX6 (LIM Homeobox 6)^+^ interneurons in the CP of the neocortex at E18.5 cortex (Squarzoni et al., 2014; Figure 3). Microglial depletion or LPS-induced MIA disrupted the precise localization of LHX6^+^ interneurons in layer V, causing premature neocortical entry and broader dispersion. These processes are regulated through the CX3C motif chemokine receptor 1 (CX3CR1) and DNAX-activating protein of 12 kDa (DAP12) signaling pathways (Squarzoni et al., 2014). Yu et al. (2022) demonstrated that maternal IL-17a signaling, induced by prenatal administration of Poly(I:C) at E12.5, led to the downregulation of G protein-coupled receptor 56 (GPR56) expression in microglia collected from E14.5 mouse brains (Yu et al., 2022). Deletion of GPR56 impaired the proliferation of NPCs destined to form parvalbumin (PV)-expressing interneurons, recapitulating the reduced number of PV interneurons and the behavioral abnormalities observed following MIA. Importantly, restoring GPR56 expression after MIA induction partially rescued PV interneuron deficits and ASD-like behaviors in mice at P21 (Yu et al., 2022).

Together, these findings suggest that prenatal immune challenges disrupt the development of inhibitory circuits, likely due to microglial dysfunction, thereby increasing the risk of behaviors associated with NDDs.

### 5.4 Physiological and MIA-induced effects on gliogenesis

The CNS contains various types of glial cells, including astrocytes, oligodendrocytes, and microglia (Barres, 1999; Allen and Lyons, 2018). Astrocytes are the most abundant glial cell type and perform a wide range of functions essential for brain activity, whereas oligodendrocytes form the myelin sheath around neuronal axons, which is critical for the efficient transmission of neuronal signals (Domingues et al., 2016).

Previous studies reported that embryonic microglia affect gliogenesis in the developing brain. Nakanishi et al. (2007) reported that microglia-derived factors, such as IL-6 and leukemia inhibitory factor (LIF), promote the differentiation of NSCs/NPCs, isolated from the SVZ of E16 rat embryos, into astrocytes (Figure 2). Another study revealed that a subpopulation of activated microglia accumulates near the third ventricle and interact with Olig2^+^ progenitors, which give rise to oligodendrocytes and astrocytes in the later-stage hypothalamus (Marsters et al., 2020). By E11.5, microglia invade the tuberal hypothalamus, and by E15.5, they cluster alongside Olig2^+^ progenitors near the third ventricle. In a microglial depletion model using PLX5622, Olig2^+^ cell migration from the VZ was delayed. It also disrupted the maturation and migration of oligodendrocyte progenitors (OPCs) in the gray matter during the embryonic and early postnatal stages (Marsters et al., 2020). Microglia-derived C-C motif chemokine ligand (CCL) 2 (CCL2) and C-X-C motif chemokine ligand 10 (CXCL10) influence neuronal differentiation at the expense of astrocyte differentiation. Since CCL2 released from microglia promotes an oligodendrocyte fate, microglia near the third ventricle, which adopt an activated phenotype, are crucial for proper oligodendrocyte maturation and influence gliogenesis in the developing tuberal hypothalamus. Similar activation of microglia is observed in the forebrain SVZ, highlighting their role in regulating glial progenitor fate decisions in the embryonic brain (Marsters et al., 2020).

While the involvement of microglia in gliogenesis during the embryonic period has been reported, the impact of MIA remains largely unclear. Further research is needed to elucidate how MIA affects microglial functions in gliogenesis.

### 5.5 Physiological and MIA-induced effects on microglial mobility and motility

As mentioned above, the distribution patterns of microglia within the brain parenchyma change in a stage-dependent manner (Swinnen et al., 2013; Hattori, 2021). The transient absence of microglia from the CP at E15–E16, caused by their bidirectional migration at E14, has been shown to be important for the proper neuronal subtype maturation (Hattori et al., 2020). In addition, microglia actively move throughout the developing brain during embryonic stages, with particularly rapid movement observed at E14 (Swinnen et al., 2013). This mobility has been shown to depend on the interaction between CXCL12 and CXCR4 (Hattori and Miyata, 2018). Specifically, inhibiting microglial motility by administering a CXCR4 antagonist (AMD3100) led to a reduction in TBR2^+^ IPs and an increase in PAX6^+^ NSCs (Hattori and Miyata, 2018). Blood vessels are also believed to contribute to local microglial migration within the embryonic brain. Microglia express α5β1 integrin, the receptor for fibronectin, and are found surrounding blood vessels, which are known to express fibronectin during development (Grossmann et al., 2002; Milner and Campbell, 2003; Gama Sosa et al., 2021; Smolders et al., 2017). A previous study reported that blood vessels regulate microglial migration via the interaction of α5β1 integrin and fibronectin in a stage-dependent manner in the embryonic mouse brain (Smolders et al., 2017). At E13.5, α5β1 integrin facilitates active microglial migration, whereas at E15.5 and E17.5, microglial migration slows due to decreased cortical fibronectin production and reduced α5β1 integrin expression on microglia (Smolders et al., 2017). Overall, the microglial distribution and migration in the developing CNS are spatiotemporally regulated by multiple mechanisms.

A recent study investigated the mobility and motility of microglia in MIA states. Ozaki et al. (2020) reported that MIA induced by Poly(I:C) injection at E12 or E15 increased IL-6 expression in the maternal liver, placenta and embryonic microglia (Figure 3). Microglial morphology in both fetal (E18) and postnatal (P10) brains remained largely unchanged, with only minor alterations observed in other biochemical characteristics. However, live imaging of acute brain slice culture revealed that MIA induced at E12 or E15 resulted in altered process motility of embryonic microglia (E18) in the somatosensory cortex. *In vivo* imaging using two-photon microscopy revealed that these changes persisted into the postnatal (P10) stage but in opposing directions, as the speed of microglial process tip movement was increased at E18 but decreased at P10, suggesting that these abnormal characteristics may result in behavioral deficits (Ozaki et al., 2020).

While research on the motility of fetal microglia in MIA models remains scarce, a previous study has examined its impact on CAM migration (Cui et al., 2020). Cui et al. (2020) showed that CAMs in the choroid plexus of the fourth ventricle exhibited abnormal motility and mobility following MIA induction with Poly(I:C), as observed through two-photon *in vivo* imaging. They demonstrated that MIA induces the accumulation of phagocytic macrophages in this region via elevated chemotactic CCL2/C-C motif chemokine receptor 2 (CCR2) signaling at the embryonic choroid plexus–cerebrospinal fluid interface (Cui et al., 2020).

Collectively, these studies suggest that maternal immune status is closely linked to the behavior of brain macrophages, indicating their vulnerability during gestation. It is essential to carefully observe and investigate how fetal microglia respond in pathological conditions and whether they may be linked to the subsequent risk of NDDs in offspring.

## 6 Single-cell transcriptional changes in microglia in response to MIA

In addition, recent single-cell analyses have revealed the diverse properties of microglia. In the physiological conditions, microglia tend to show more heterogenous, diverse phenotypes in the early developmental stage compared to their adult counterparts. For example, Matcovitch-Natan et al. (2016) transcriptionally profiled gene expression and analyzed epigenetic signatures of microglia at the single-cell level during mouse development, identifying three distinct temporal stages: early microglia (until E14), pre-microglia (from E14 to a few weeks after birth), and adult microglia (from a few weeks after birth onward). They further characterized the differential regulatory elements in each developmental phase. They further found that microglia from germ-free mice exhibited dysregulation of genes associated with the adult phase and immune response, indicating microbiome influence on microglial development (Matcovitch-Natan et al., 2016). In addition, Hammond et al. (2019) analyzed RNA expression in over 76,000 microglia in mice across development, aging, and injury, and identified nine transcriptionally distinct states with unique gene sets. While several states, including chemokine-enriched inflammatory microglia, persisted or increased with age, microglial heterogeneity was greatest in early life (Hammond et al., 2019).

Human fetal microglia exhibit extensive transcriptional similarities to developing mouse microglia. The transition from a heterogeneous, proliferative state to a more homogeneous, homeostatic, and immune-sensing phenotype is also observed in humans. Single-cell RNA sequencing of 23 human fetuses ranging from GW9–18 revealed that fetal microglia initially form a heterogeneous pool but gradually acquire a homeostatic phenotype with increasing GWs (Kracht et al., 2020). Moreover, Assay for Transposase-Accessible Chromatin (ATAC)-sequencing data showed that enhanced chromatin accessibility in older fetal microglia enables the activation of gene programs essential for environmental sensing, synaptic pruning, phagocytosis, and tissue supportive functions, suggesting the emergence of immune-sensing microglia during early fetal development. As development proceed, microglia start to mature and increasingly resemble adult microglia with CNS-surveilling properties with immune-sensing competent phenotype. This might render the developing human CNS vulnerable to environmental perturbations during early pregnancy (Kracht et al., 2020).

Single-cell transcriptome analyses have also showed that this diversity fluctuates in response to MIA. Matcovitch-Natan et al. (2016) examined microglia from both newborn and adult offspring of mice whose mothers were injected with Poly(I:C) at E14. Their analysis revealed that MIA had the pronounced effect on microglia in newborns, causing a transcriptional shift toward a more advanced developmental state. Notably, in adult microglia, they observed significantly fewer differentially expressed developmental genes compared to microglia from newborns, suggesting that the transcriptional profile of Poly(I:C)-exposed mice eventually was realigned with that of the normal phenotype in adulthood. These findings indicate that transient disruptions during microglia development could have lasting consequences for brain function later in life (Matcovitch-Natan et al., 2016).

Hayes et al. (2022) also investigated the impact of MIA on the offspring’s immune response. They injected Poly(I:C) into the dam at E9.5 and later administered either LPS or saline to the adult offspring. Single-cell RNA sequencing of isolated microglia revealed that, in the MIA model, offspring microglia exhibited a long-term decrease in immune reactivity throughout development compared to controls (saline-treated). This diminished immune response was accompanied by alterations in chromatin accessibility and reduced transcription factor occupancy in open chromatin. Moreover, microglia from the frontal cortex and striatum of MIA offspring both displayed a blunted immune response relative to control microglia, with the effect being more pronounced in the striatum than in the frontal cortex. Notably, they found that MIA does not generate a distinct subpopulation of microglia but rather reduces the contribution of microglia to inflammatory states (Hayes et al., 2022).

Another study has discussed the potential association between the reduced immune responsiveness observed in adult microglia following prenatal MIA exposure. In the study by Ostrem et al. (2024) MIA was induced at E12.5 using Poly(I:C), or saline was administered as a control. Subsequently, single cell RNA-sequencing was performed on microglia isolated via FACS from the presumptive somatosensory cortex at E13.5 and E15.5. The analysis revealed an upregulation of inflammation-related genes such as *Ccl2*, JunD Proto-Oncogene (*Jund*), interferon stimulated gene 15 (*Isg15*), and C-type lectin domain family 12 member A (*Clec12a*) at E13.5 following MIA exposure. However, the widespread increase in gene expression associated with microglial activation observed at E13.5 was not maintained at E15.5. Instead, gene ontology (GO) term analysis indicated an enrichment of pathways related to “aging” and “ATP synthesis coupled electron transport.” These findings suggest that the microglial change their response to MIA over time (Ostrem et al., 2024). Furthermore, an investigation into persistent gene expression changes in microglia from juvenile (P14) mice exposed to MIA revealed a downregulation of the transcription factor hairy and enhancer of split 1 (*Hes1*) (Ostrem et al., 2024). Since the reduced expression of Hes1 has also been reported in microglia treated with anti-inflammatory compounds (Yao et al., 2022), the data indicate that microglia may exhibit a prolonged compensatory anti-inflammatory response for several weeks following MIA exposure (Ostrem et al., 2024).

Recent advances in single-cell transcriptomics have provided key insights into microglial diversity in both humans and mice. Notably, microglia exhibit a high degree of heterogeneity early in development, particularly during the embryonic stage, and this diversity gradually decreases as they mature, becoming more homogeneous over time until young adult stage. Extensive studies in rodent models have revealed that MIA exposure alters the transcriptional profile of fetal microglia. Specifically, MIA accelerates microglial maturation, potentially driving them toward an adult-like profile at an earlier stage. Furthermore, traces of immune activation induced by MIA may persist into postnatal and adult offspring, leading to a long-lasting reduction in immune responsiveness to secondary inflammatory stress. These findings suggest that MIA exposure can have profound and enduring effects on brain function and disease susceptibility. If microglial stage-specific functions are disrupted by genetic or environmental perturbations, this may lead to a loss of brain homeostasis, potentially contributing to NDDs.

## 7 Discussion

In terms of the ontogeny of microglia and their process of colonizing the brain during development, emerging evidence has been gradually clarifying the similarities and differences between humans and mice. Moreover, epidemiological studies in humans have suggested that, in addition to genetic factors, MIA is associated with an increased risk of NDDs in offspring. However, due to the difficulty of conducting detailed mechanistic analyses and causal investigations in humans, the need for animal models—primarily MIA models using rodents—has increased, and research in this area has been steadily advancing. Notably, recent reports indicate that microglia in the fetal mouse brain are likely to be highly responsive to MIA, exerting significant effects on surrounding cells. Thus, this review focused on previous studies that examined the physiological functions of microglia and the alterations induced by MIA during the embryonic stage.

The MIA animal models primarily mimic viral or bacterial infections using Poly(I:C) or LPS, respectively. Importantly, however, MIA encompasses a wide range of inflammation types. In addition to infections, maternal diet, stress, and external factors are also known to trigger immune responses. For example, previous studies have investigated the effects of a maternal diet on fetal brain microglial properties (Bordeleau et al., 2020; Ceasrine et al., 2022). A maternal high-fat diet altered microglial morphology and increased microglial interactions with astrocytes in the hippocampal CA1 region, particularly in male offspring (Bordeleau et al., 2020). Another study reported that in male offspring exposed to a maternal high-fat diet, increased macrophage Toll-like receptor 4 signaling leads to excessive microglial phagocytosis of serotonin (5-HT) neurons in the developing dorsal raphe nucleus. This reduces 5-HT bioavailability, resulting in sex-specific behavioral outcomes in the offspring (Ceasrine et al., 2022). In addition, gestational exposure to environmental stress and socioeconomic stressors also impacts microglial function (Block et al., 2022; Rosin et al., 2021). Block et al. (2022) demonstrated that prenatal exposure to intermittent diesel exhaust particle (DEP) instillations, mimicking chronic air pollution, disrupted microglial function in the anterior cingulate cortex, which is a critical region for processing social networks, in male offspring during early postnatal development. This leads to lasting behavioral deficits and alterations in brain network activity associated with social interactions (Block et al., 2022). Maternal cold stress also impacts on fetal brain microglial function through induction of C-C motif chemokine ligand (CCL) 3 (CCL3) and CCL4 secretion, exclusively in male fetuses. (Rosin et al., 2021). In adulthood, male offspring exposed to maternal cold stress exhibited microglia-dependent changes in social behavior. Collectively, the diverse models of MIA—including infections, dietary influences, and environmental or psychosocial stressors—highlight the significant impacts of MIA and its downstream effects on offspring neurodevelopment, mediated through microglial activity. Notably, male offspring appear to be particularly vulnerable to microglia-mediated disruptions in neural circuit formation (Bergdolt and Dunaevsky, 2019; Bordeleau et al., 2020; Ceasrine et al., 2022; Rosin et al., 2021).

On the other hand, research on MIA has shown varying results, including cases where effects are present, absent, or manifest differently. These discrepancies can depend on the conditions under which MIA is induced, such as the number of inductions, timing, and method of administration, as well as the animal species used and the timing of analysis (Woods et al., 2023; Hameete et al., 2021). Notably, the source, molecular weight, and endotoxin levels of Poly(I:C) influence maternal and prenatal outcomes, highlighting their importance in MIA models (Kowash et al., 2019; Mueller et al., 2019). To complicate matters further, it has also been reported that the intrauterine position influences behavioral outcomes following MIA (Schaer et al., 2024). Therefore, it is crucial to provide a precise and detailed description of the procedures, including how MIA was induced and the methods used for analysis. Moreover, as sex differences have been observed in previous analyses, it seems necessary to investigate such differences in greater detail during the embryonic stage as well (Bergdolt and Dunaevsky, 2019; Bordeleau et al., 2020; Ceasrine et al., 2022; Rosin et al., 2021; Kalish et al., 2021).

In addition, recent advances in single-cell transcriptomics have provided new insights into microglial diversity (Matcovitch-Natan et al., 2016; Hammond et al., 2019; Kracht et al., 2020; Hayes et al., 2022; Ostrem et al., 2024). In both humans and mice, microglia exhibit significant heterogeneity early in development, particularly during the embryonic stage (Hammond et al., 2019; Kracht et al., 2020). With advancements in single-cell technologies, the effects of MIA have been extensively studied in rodent models, revealing that fetal microglial transcriptional profiles are altered by MIA exposure. Specifically, MIA appears to accelerate microglial maturation, shifting their profile toward that of adult microglia at an earlier stage (Matcovitch-Natan et al., 2016). Furthermore, studies suggest that MIA leaves lasting immunological imprints, potentially leading to reduced immune responsiveness to secondary inflammatory stress in postnatal and adult offspring (Hayes et al., 2022). Therefore, gaining a deeper understanding of the impact of MIA on microglial properties from the embryonic stage through postnatal development and into adulthood will provide valuable insights into the pathophysiology of NDDs.

Modeling the effects of prenatal risk factors still relies heavily on rodent exposure studies (Meyer, 2023). Studying these effects in humans remains exceptionally challenging due to the difficulty of conducting long-term follow-up studies and the limited accessibility of relevant samples. However, recent advances in organoid research using human induced pluripotent stem cells (iPS cells) may enable attempts to examine the impact of MIA. Cerebral organoids are self-organizing, brain-like structures that provide a valuable tool for studying human brain development (Lancaster et al., 2013) and diseases, including NDDs (Sarieva et al., 2023). However, to date, conventional cerebral organoids are derived from ectodermal lineage cells, and most models lack other germ layer-derived cells, including microglia. Recently, efforts have been made to incorporate microglia into organoids. Several groups have described protocols for generating microglia-containing organoids, either by co-culturing organoids with differentiated microglial cells (Popova et al., 2021; Park et al., 2023), microglial progenitors (Fagerlund et al., 2021), or through spontaneous formation (Ormel et al., 2018; Cakir et al., 2022). A recent study investigated the inflammatory response using cerebral organoids with integrated microglia (COiMg) (Buonfiglioli et al., 2025). The addition of interferon-gamma (IFN-γ) induced significant transcriptional and structural changes in the cerebral organoids, and these changes appeared to be modulated by the presence of microglia. Specifically, IFN-γ was found to alter the expression of ASD-related genes (Buonfiglioli et al., 2025).

Overall, future research must adopt a comprehensive developmental perspective to investigate how MIA alters microglial diversity and functional properties across various time points and how long these changes persist. It is also crucial to determine how these alterations impact brain development and which cell populations play the most critical roles in these processes using single-cell-based analyses. On the other hand, spatial information and the molecular mechanisms governing these complex processes cannot be fully elucidated through transcriptomic data alone. Thus, future studies may need to incorporate spatial transcriptomics, and high-resolution imaging technologies including *in vivo* live imaging, and to gain deeper insights into these intricate mechanisms. MIA itself is highly complex, encompassing not only the infection models such as Poly(I:C) or LPS administration, which were the focus of this review, but also a wide range of other infectious agents, maternal stress, and external environmental factors. A more comprehensive understanding of these effects will require the use of diverse animal models.

Currently, most MIA research relies on rodent models; however, expanding studies to other species, including primates such as marmosets and monkeys, could further enhance our understanding of human pathophysiology. Furthermore, in the context of human disease modeling, advancements in organoid research using human iPS cells may serve as a powerful tool for elucidating the roles of microglia and their perturbations in human brain development. In summary, future research should aim to prevent or mitigate MIA-induced neurodevelopmental abnormalities by elucidating microglial properties during critical developmental periods. Such efforts will be contribute to the early diagnosis of pathological conditions and the development of therapeutic strategies in the future.
